# Stock price prediction using principal components

**DOI:** 10.1371/journal.pone.0230124

**Published:** 2020-03-20

**Authors:** Mahsa Ghorbani, Edwin K. P. Chong

**Affiliations:** 1 PhD Student, Department of Systems Engineering, Colorado State University, Fort Collins, Colorado, United States of America; 2 Professor, Department of Electrical and Computer Engineering, Colorado State University, Fort Collins, Colorado, United States of America; The Bucharest University of Economic Studies, ROMANIA

## Abstract

The literature provides strong evidence that stock price values can be predicted from past price data. Principal component analysis (PCA) identifies a small number of principle components that explain most of the variation in a data set. This method is often used for dimensionality reduction and analysis of the data. In this paper, we develop a general method for stock price prediction using time-varying covariance information. To address the time-varying nature of financial time series, we assign exponential weights to the price data so that recent data points are weighted more heavily. Our proposed method involves a dimension-reduction operation constructed based on principle components. Projecting the noisy observation onto a principle subspace results in a well-conditioned problem. We illustrate our results based on historical daily price data for 150 companies from different market-capitalization categories. We compare the performance of our method to two other methods: Gauss-Bayes, which is numerically demanding, and moving average, a simple method often used by technical traders and researchers. We investigate the results based on mean squared error and directional change statistic of prediction, as measures of performance, and volatility of prediction as a measure of risk.

## Introduction

Predicting future stock price values is a very challenging task. There is a big body of literature on different methods and different predictors to incorporate into those methods to predict the future values as closely as possible. The literature provides strong evidence that past price/return data can be used to predict future stock prices. Some studied have found significant auto-correlation for returns over a short period of time. French and Roll find negative correlation for individual securities for daily returns [1]. Some other studies show there is a positive correlation for returns over the period of weeks or months [2]. Studies also demonstrate stock return correlation over the period of multiple months or years. Fama and French report that the auto-correlation is stronger for longer periods, three to five years, compared to daily or weekly periods [3]. Cutler et al. report positive auto-correlation over the horizon of several months and negative auto-correlation over the horizon of three to five years [4]. There are some other studies that also show correlation in stock returns over a multiple year interval [5, 6] which all confirm that price/return values are predictable from past price/return values.

Bogousslavsky shows that trading by investors with heterogeneous rebalancing horizons can give rise to autocorrelation in the returns at different frequencies [7]. Chowdhury et al. investigate the autocorrelation structure of seven Gulf Cooperation Council (GCC) stock markets. All the markets except for Dubai and Kuwait show significant first-order autocorrelation of returns. They also find that autocorrelation between weekdays is usually larger than that between the first and last trading days of the week [8]. Li et al. study the nonlinear autoregressive dynamics of stock index returns in seven major advanced economies (G7) and China using the quantile autoregression model. For the stock markets in the seven developed economies, the autoregressive parameters generally follow a decreasing pattern across the quantiles with significant portions outside the ordinary least squares estimate intervals [9]. Another study investigates the autocorrelation structure of stock and portfolio returns in the unique market setting of Saudi Arabia [10]. Their results show that there is significantly positive autocorrelation in individual stock and market returns. Another study applies the threshold quantile autoregressive model to study stock return autocorrelations in the Chinese stock market [11]. They report negative autocorrelations in the lower regime and positive autocorrelations in the higher regime.

Other fundamental or macroeconomic factors can also be used in predicting future stock price values. Macroeconomic factors such as interest rates, expected inflation, and dividend can be used in stock return predictions models [3, 12]. Also fundamental variables such as earnings yield, cash flow yield, size and book to market equity [13, 14] have been found to have estimation power in predicting future price/return values.

Silvennoinen and Teräsvirta report correlation between individual U.S. stocks and the aggregate U.S. market [15]. Dennis et al. study the dynamic relation between daily stock returns and daily volatility innovations, and they report negative correlations [16]. Another study investigates the effect of common factors on the relationship among stocks and on the distribution of the investment weights for stocks [17]. They report that market plays a dominant role in both structuring the relationship among stocks and in constructing a well-diversified portfolio. Dimic et al. examine the impact of global financial market uncertainty and domestic macroeconomic factors on stock–bond correlation in emerging markets [18]. In another study, the focus is analyzing the impact of oil price shocks on the interactions of oil-stock prices [19]. The results show that negative changes in oil prices have a significant impact on the stock market.

In this paper, we describe a general method for predicting future stock price values based on historical price data, using time-varying covariance information. When the number of observations is large compared to the number of predictors, the maximum-likelihood covariance estimate [20] or even the empirical covariance is a good estimate of the covariance of the data, but that is not always the case. When the number of observations is smaller than the matrix dimension, the problem is even worse because the matrix is not positive definite [21]. This problem, which happens quite often in finance, gives rise to a new class of estimators such as shrinkage estimators. For example Ledoit and Wolf, shrink the sample covariance towards a scaled identity matrix using a shrinkage coefficient that minimizes the mean squared error of the prediction [22]. Some other studies in this field include [23–25]. In our numerical evaluations in this paper we have sufficient empirical data to reliably track the covariance matrix over time.

Momentum-based forecasting relies on prices following a trend, either upwards or downwards. Based on the assumption that trends like this exist and can be exploited, momentum is used as a heuristic rule for forecasting and is probably the most popular technical indicator used by traders; in particular, the method of *Direction Movement Index (DMI)*, due to Wilder [26]. This kind of heuristic is a special case of pattern-based forecasting, where, in the case of momentum, the pattern is simply the upward or downward trend. Our method is a systematic method to capture arbitrary patterns, not just upward or downward trends. Indeed, we compute prevalent patterns in the form of eigenvectors (or “eigen-patterns”) of the local covariance matrix. As such, we are able to exploit more general patterns that are prevalent (but not necessary known beforehand) in price time series.

The mean squared error (MSE) measures the distance between predicted and real values and is a very common metric to evaluate the performance of predictive methods [27]. Multivariate conditional mean minimizes the mean squared error [28] and is a good estimator for future price values. However, numerical results using this method cannot always be trusted because of associated ill-conditioning issues. In this paper we introduce a method with similar estimation efficiency that does not suffer from this issue.

Principal component analysis (PCA), which is a method for dimensionality reduction of the data, is used in different fields such as statistical variables analysis [29], pattern recognition, feature extraction, data compression, and visualization of high dimensional data [30]. It also has various application in exploring financial time series [31], dynamic trading strategies [32], financial risk computations [32, 33], and statistical arbitrage [34]. In this work, we implement PCA in estimating future stock price values.

Yu et al. introduce a machine-learning method to construct a stock-selection model, which can perform nonlinear classification of stocks. They use PCA to extract the low-dimensional and efficient information [35]. In another study, three mature dimensionality reduction techniques, PCA, fuzzy robust principal component analysis, and kernel-based PCA, are applied to the whole data set to simplify and rearrange the original data structure [36]. Wang et al. present a stochastic function based on PCA developed for financial time-series prediction [37]. In another study, PCA is applied to three subgroups of stocks of the Down Jones Industrial (DJI) index to optimize portfolios [38]. Narayan et al. apply PCA to test for predictability of excess stock returns for 18 emerging markets using a range of macroeconomic and institutional factors [39].

Factor analysis is a technique to describe the variability of observed data through a few factors and is in some sense similar to PCA. There is a long debate in the literature on which method is superior [40, 41]. Factor analysis begins with the assumption that the data comes from a specific model where underlying factors satisfy certain assumptions [42]. If the initial model formulation is not done properly, then the method will not perform well. PCA on the other hand involves no assumption on the form of the covariance matrix. In this paper, we focus on developing an algorithm that can ultimately be used in different fields without prior knowledge of the system, and therefore PCA is the method of choice. In the case study presented in the following section, although only price data is used, it would have been also possible to include multiple predictors to estimate futures values of stock prices.

Our method bears some similarity with subspace filtering methods. Such methods assume a low-rank model for the data [43]. The noisy data is decomposed onto a signal subspace and noise based on a modified singular value decomposition (SVD) of data matrices [44]. The orthogonal decomposition can be done by an SVD of the noisy observation matrix or equivalently by an eigenvalue decomposition of the noisy signal covariance matrix [43].

We compare the performance of our proposed methods in terms of MSE and directional change statistic. Stock-price direction prediction is an important issue in the financial world. Even small improvements in predictive performance can be very profitable [45]. Directional change statistic calculates whether our method can predict the correct *direction* of change in price values [46]. It is an important evaluation measure of the performance because predicting the direction of price movement is very important in some market strategies.

Another important parameter that we are interested in is standard deviation, one of the key fundamental risk measures in portfolio management [47]. The standard deviation is a statistical measure of volatility, often used by investors to measure the risk of a stock or portfolio.

As mentioned above, in this paper we focus on forecasting stock prices from daily historical price data. In Section, we introduce our technical methodology, and in particular estimation techniques using covariance information. In Section, we describe our method for processing the data and estimating the time-varying covariance matrix from empirical data, including data normalization. We also demonstrate the performance of our method.

## Theoretical methodology

### Estimation techniques

In this section we introduce a new computationally appealing method for estimating future stock price values using covariance information. The empirical covariance can be used as an estimate of the covariance matrix if enough empirical data is available, or we can use techniques similar to the ones introduced in the previous section, though the time-varying nature of the covariance must be addressed.

Suppose that we are given the stock price values for *M* days. Our goal is to predict company stock prices for *M* + 1 to *N* trading days, using the observed values of the past consecutive *M* days. The reason for introducing *N* will be clear below.

#### Gauss-Bayes or conditional estimation of *z* given *y*

Suppose that *x* is a random vector of length *N*. Let *M* ≤ *N* and suppose that the first *M* data points of vector *x* represent the end-of-day prices of a company stock over the past *M* consecutive trading days. The multivariate random vector *x* and can be partitioned in the form

$$\begin{array}{c} x=[\begin{array}{cc} y & z \end{array}]. \end{array}$$ (1)

Let random vector *y* represent the first *M* data points and *z* the price of the next *N* − *M* days in the future. We wish to estimate *z* from *y*.

The covariance matrix for the random vector *x* can be written as

$$\begin{array}{c} \Sigma_{xx}=[\begin{array}{cc} \Sigma_{yy} & \Sigma_{yz}\\ \Sigma_{zy} & \Sigma_{zz} \end{array}], \end{array}$$ (2)

where Σ_*yy*_ is the covariance of *y* and Σ_*zz*_ is the covariance of *z*. Assuming that *y* and *z* are jointly normally distributed, knowing the prior distribution of *x* = [*y*, *z*], the Bayesian posterior distribution of *z* given *y* is given by

$$\begin{array}{cc} {\hat{z}}_{z|y} & =\Sigma_{zy}\Sigma_{yy}^{-1}y\\ {\hat{\Sigma }}_{z|y} & =\Sigma_{zz}-\Sigma_{zy}\Sigma_{yy}^{-1}\Sigma_{yz}. \end{array}$$ (3)

The ${\hat{\Sigma }}_{z|y}$ matrix, representing the conditional covariance of *z* given *y*, is also called the Schur complement of Σ_*yy*_ in Σ_*xx*_. Note that the posterior covariance does not depend on the specific realization of *y*.

The Gauss-Bayes point estimator for the price prediction, the conditional mean ${\hat{z}}_{z|y}$, minimizes the mean squared error of the estimate in the Gaussian case [28]. Moreover, in the Guassian case, for a specific observation *y*, the inverse of the conditional covariance is the Fisher Information matrix associated with estimating *z* from *y*, and therefore ${\hat{\Sigma }}_{z|y}$ is the lower bound on the error covariance matrix for any unbiased estimator of *z* [28].

The same set of equations arise in Kalman’s filtering. Kalman’s own view of this process is as a completely deterministic operation [48], and does not rely on assuming normality. Although the point estimator ${\hat{z}}_{z|y}$ is optimal in term of mean squared error, in practice there are numerical complications involved in this method: The matrix Σ_*yy*_ is typically not well conditioned, so the numerical calculation of $\Sigma_{yy}^{-1}$ cannot always be trusted. To overcome this problem, we propose a better conditioned estimator, which has a behavior close to Gauss-Bayes.

#### Principal components and estimation in lower dimension

Principal component analysis (PCA) is a well-established mathematical procedure for dimensionality reduction of data and has wide applications across various fields. In this work, we consider its application in forecasting stock prices.

Consider the singular value decomposition (SVD) of Σ_*xx*_:

$$\begin{array}{c} \Sigma_{xx}=VSV^{\prime }, \end{array}$$ (4)

where *S* is a diagonal matrix of the same dimension as *x* with non-negative diagonal elements in decreasing order, and *V* is a unitary matrix (*VV*′ = *I*_*N*_). The diagonal elements of *S* are the eigenvalues of Σ_*xx*_.

In general, the first few eigenvalues account for the bulk of the sum of all the eigenvalues. The “large” eigenvalues are called the principal eigenvalues. The corresponding eigenvectors are called the principal components.

Let *L* < *N* be such that the first *L* eigenvalues in *S* account for the bulk part (say 85% or more) of the sum of the eigenvalues. Let *V*_*L*_ be the first *L* columns of unitary matrix *V*. Then the random vector *x* is approximately equal to the linear combination of the first *L* columns of *V*:

$$\begin{array}{c} x\approx V_{L}\alpha , \end{array}$$ (5)

where *α* is a random vector of length *L*. Because *L* is a small number compared to *N*, Eq (5) suggests that a less “noisy” subspace with a lower dimension than *N* can represent most of the information. Projecting onto this principle subspace can resolve the ill-conditioned problem of Σ_*yy*_. The idea is that instead of including all eigenvalues in representing Σ_*xx*_, which vary greatly in magnitude, we use a subset which only includes the “large” ones, and therefore the range of eigenvalues is significantly reduced. The same concept is implemented in speed signal subspace filtering methods, which are based on the orthogonal decomposition of noisy speech observation space onto a signal subspace and a noise subspace [43]. Let *V*_*M*,*L*_ be the first *M* rows and first *L* columns of *V*. We have

$$\begin{array}{c} y=V_{M,L}\alpha +\text{Noise}. \end{array}$$ (6)

Mathematically resolving noisy observation vector *y* onto the principle subspace can be written as a filtering operation in the form of

$$\begin{array}{c} w=Gy, \end{array}$$ (7)

where *G* is given by

$$\begin{array}{c} G={\left(V_{M,L}^{{}^{\prime }}V_{M,L}\right)}^{-1}V_{M,L}^{{}^{\prime }}. \end{array}$$ (8)

The vector *w* is actually the coordinates of the orthogonal projection of *y* onto the subspace equal to the range of *V*_*M*,*L*_. We can also think of *w* as an estimate of *α* based on least squares. Substituting *y* by *w* in (3) leads to a better conditioned set of equations:

$$\begin{array}{cc} {\hat{z}}_{z|w} & =\Sigma_{zw}\Sigma_{ww}^{-1}w\\ {\hat{\Sigma }}_{z|w} & =\Sigma_{zz}-\Sigma_{zw}\Sigma_{ww}^{-1}\Sigma_{wz}, \end{array}$$ (9)

because the condition number of Σ_*ww*_ is much lower than that of Σ_*yy*_, as we will demonstrate later. In (9) we have

$$\begin{array}{c} \Sigma_{zw}=E[zw^{{}^{\prime }}]=\Sigma_{zy}G^{{}^{\prime }}, \end{array}$$ (10)

and

$$\begin{array}{c} \Sigma_{ww}=E[ww^{{}^{\prime }}]=G\Sigma_{yy}G^{{}^{\prime }}. \end{array}$$ (11)

If the posterior distribution of *z* estimated based on (9) has a similar behavior to the distribution estimated by (3), it can be considered a good substitute for the Gauss-Bayes method. Our numerical results demonstrate that this is indeed the case, which we will show in Section.

#### Moving average

Technical traders and investors often use technical trading rules, and one of the most popular methods used by technical traders and researchers are the moving average (MA) rules [49, 50]. Satchell investigates the reason general MA trading rules are widely used by technical analysts [51]. He shows that autocorrelation amplification is one of the reasons such trading rules are popular. Using simulated results, we show that the MA rule may be popular because it can identify the price momentum and is a simple way of assessing and exploiting the price autocorrelation without necessarily knowing its precise structure. Moving average, which is the average of prices over a period of time, is probably the simplest estimator for *z*:

$$\begin{array}{cc} {\hat{z}}_{\text{MA}} & =\frac{1}{K_{MA}}\sum_{i=N-K_{MA}+1}^{N}x_{i} \end{array}$$ (12)

where the quantity *K*_*MA*_ is the number of data points included to calculate the average, and ${\hat{z}}_{MA}$ is the average of the most recent *K*_*MA*_ price values.

There are different possible values of *K*_*MA*_ for calculating the average, from short to medium to long term periods. Here we use periods of 10 and 50 days, which are typical short and midterm values used in the literature. We will use the moving average estimator for comparison purposes, as we will see in Section below.

**Fig 1:**
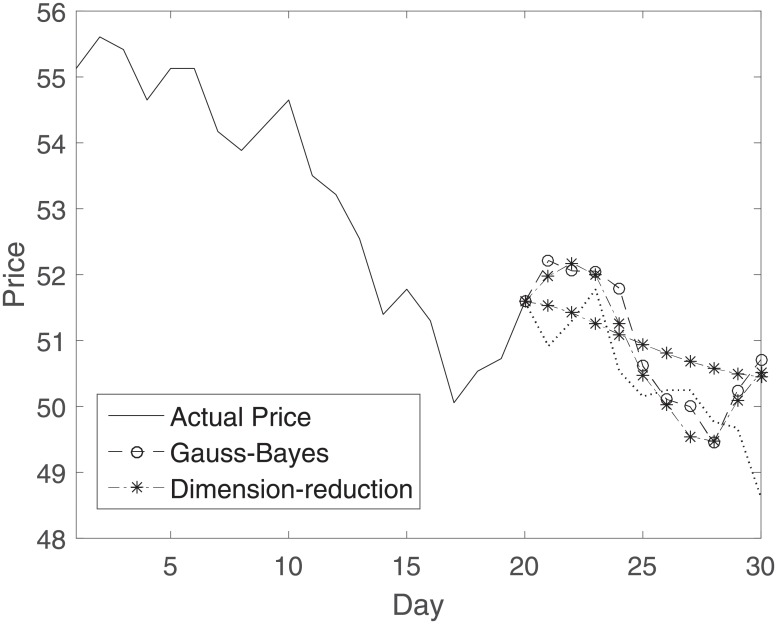
Predicting price for *M* + 1 to *N* days, actual price: Solid line, GB: −*o*−, RD: −*− (two lines, one for a small value of L, and one for a relatively large number).

Fig 1 shows an example of our stock predictions. Assume that we are given the price values for the past 20 days (*M* = 20), and we want to use those values to predict the future prices over the next 10 business days, from day *M* + 1 to day *N* (*N* = 30). In our reduced-dimension technique, we can get a relatively smooth plot of the predicted value for a relatively small *L*, to a plot almost the same as Gauss-Bayes, for larger values of *L*, as we can can see in Fig 1.

### Performance metrics

#### Mean squared error

To compare the performance of the methods described above, we evaluate the expected value of the squared error between the actual and estimated values. The mean squared error of an estimate $\hat{z}$ is given by:

$$\begin{array}{c} MSE=E[\begin{array}{c} \|z-\hat{z}{\|}^{2} \end{array}]=E[\begin{array}{c} \|z{\|}^{2} \end{array}]+E[\begin{array}{c} \|\hat{z}{\|}^{2} \end{array}]-2E[\begin{array}{c} \left\|z^{\prime }\hat{z}\right\| \end{array}]. \end{array}$$

The MSE can be expressed in terms of the covariance matrices in (2), by substituting the appropriate form of $\hat{z}$. Alternatively, the mean squared error of an estimator $\hat{z}$ can be written in terms of the variance of the estimator plus its squared bias. The conditional MSE given *x* is written as

$$\begin{array}{c} MSE_{\hat{z}|z}=E[\begin{array}{c} \|z-\hat{z}{\|}^{2}|z \end{array}]=trace\left(\Sigma_{\hat{z}|z}\right)+\|E[\begin{array}{c} \hat{z}|z \end{array}]-z{\|}^{2}. \end{array}$$

The first term is called the variance, and the second term is the squared bias. The expected value of MSE over all observations is the actual MSE, which can be calculated by taking expectations on both sides:

$$\begin{array}{c} MSE=E[\begin{array}{c} E[\begin{array}{c} \|z-\hat{z}{\|}^{2}|z \end{array}] \end{array}]=trace\left(E\|\Sigma_{\hat{z}|z}\|\right)+E[\begin{array}{c} \|E[\begin{array}{c} \hat{z}|z \end{array}]-z{\|}^{2} \end{array}]. \end{array}$$ (13)

It turns out that Gauss-Bayes estimator is unbiased, which means that the second term is 0, while the proposed reduced-dimension methods is a biased estimator.

#### Directional change statistic

$$\begin{array}{c} b_{ij}=\{\begin{array}{cc} 1, & \text{if}\;\left(z_{ij}-z_{0}\right)\left({\hat{z}}_{ij}-z_{0}\right)>0\\ 0, & \text{otherwise} \end{array}. \end{array}$$ (14)

Then *D*_*j*_, the direction statistic for day *j*, averaged over *K* samples, is equal to

$$\begin{array}{cc} D_{j} & =\frac{1}{K}\sum_{i=1}^{K}b_{ij}, \end{array}$$ (15)

which is a number between 0 and 1 (the higher the better).

## Empirical methodology and results

In this section we describe how we estimate the covariance matrix based on a normalized data set, and we evaluate the performance of our method using empirical data.

### General setting

Suppose that we have *K* samples of vector data, each of length *N*, where *N* < *K*. Call these row vectors *x*_1_, *x*_2_, …, *x*_*K*_, where each $x_{i}\in R^{N}\left(i=1,\ldots ,K\right)$ is a row vector of length *N*:

$$\begin{array}{c} x_{i}=[\begin{array}{cccc} x_{i1} & x_{i2} & \cdots & x_{iN} \end{array}]. \end{array}$$ (16)

We assume that the vectors *x*_1_, *x*_2_, …, *x*_*K*_ are drawn from the same underlying distribution. We can stack these vectors together as rows of a *K* × *N* matrix:

$$\begin{array}{c} X=[\begin{array}{cccc} x_{11} & x_{12} & \cdots & x_{1N}\\ x_{21} & x_{22} & \cdots & x_{2N}\\ \cdots & \cdots & \cdots & \cdots \\ x_{K1} & x_{K2} & \cdots & x_{KN} \end{array}]. \end{array}$$

Let *M* ≤ *N* and suppose that we are given a vector $y\in R^{M}$ representing the first *M* data points of a vector we believe is drawn from the same distribution as *x*_1_, *x*_2_, …, *x*_*K*_. Again, these *M* data points represent the end-of-day prices of a company stock over the past *M* consecutive trading days. Let *z* be the price of the next *N* − *M* days in the future. We wish to estimate *z* from *y*.

Since the vector *x*_*i*_ is a multivariate random vector that can be partitioned in the form

$$\begin{array}{c} x_{i}=[\begin{array}{cc} y_{i} & z_{i} \end{array}], \end{array}$$ (17)

where *y*_*i*_ has length *M* and *z*_*i*_ has length *N* − *M*, accordingly the data matrix *X* can be divided into two sub-matrices *Y* and *Z* as follow:

$$\begin{array}{c} \begin{array}{c} X \end{array}=[\begin{array}{cc} Y & Z \end{array}]. \end{array}$$

We can think of *Y* as a data matrix consisting of samples of historical data, and *Z* as a data matrix consisting of the corresponding future values of prices.

### Normalizing and centering the data

In the case of stock-price data, the vectors *x*_1_, *x*_2_, …, *x*_*K*_ might come from prices spanning several months or more. If so, the basic assumption that they are drawn from the same distribution may not hold because the value of a US dollar has changed over time, as a result of inflation. To overcome this issue, a scaling approach should be used to meaningfully normalize the prices (we will deal with the time-varying nature of the covariance later). One such approach is presented here. Suppose that *t*_*i*_ = [*t*_*i*1_, *t*_*i*2_, …, *t*_*iN*_] is a vector of “raw” (unprocessed) stock prices over *N* consecutive trading days. Suppose that *Q* ≤ *N* is also given. Then we apply the following normalization to obtain *x*_*i*_:

$$\begin{array}{c} x_{i}=\frac{t_{i}}{t_{i}\left(Q\right)}. \end{array}$$ (18)

This normalization has the interpretation that the *x*_*i*_ vector contains stock prices as a fraction of the value on the *Q*th day, and is meaningful if we believe that the pattern of such fractions over the days 1, …, *N* are drawn from the same distribution. Note that *x*_*i*_(*Q*) = 1.

We believe normalizing the data with this method captures the pattern in the price data better than simply using return data. Although similar to return, the resulting time series still suffers from being non-stationary over time. We propose to resolve this issue by using a weighting averaging method as explained in the next section.

For the purpose of applying our method based on PCA, we assume that the vectors *x*_1_, *x*_2_, …, *x*_*K*_ are drawn from the same underlying distribution and that the mean, $\bar{x}$, is equal to zero. However because *x*_*i*_ represents price values, in general the mean is not zero. The mean $\bar{x}$ can be estimated by averaging the vector $x_{i}\in R^{N}\left(i=1,\ldots ,K\right)$,

$$\begin{array}{cc} \bar{x} & =\frac{1}{K}\sum_{i=1}^{K}x_{i}, \end{array}$$ (19)

and then this average vector is deducted from each *x*_*i*_ to center the data.

Even though this normalization makes the data stationary in the mean, since stock prices are very volatile, there is no guarantee that the covariance of the data would be stationary as well. In order to address this issue, we assign exponential weights (*γ*^0^, *γ*^1^, ⋯, *γ*^*k*^) to observations, where 0 < *γ* < 1, to emphasize the most recent periods of data. Using an exponential weighting approach to deal with volatility of financial data has been suggested in multiple studies such as [52]. For each observation *x*_*i*_, the last *K* samples prior to that observation are transformed into a Hankel matrix and normalized. Then (decreasing) exponential weights are assigned to the *K* samples and numerical results are calculated. This process, creating the matrix of data, normalizing, and assigning weights, is repeated for each observation.

To select the value of *K* we use

$$\begin{array}{c} K=\min\{k:\gamma^{k}<10^{-3}\}. \end{array}$$ (20)

## Experiments

The daily historical price data for 150 different companies from different market-capitalization categories were downloaded from finance.yahoo.com. Market capitalization is a measure of the company’s wealth and refers to the total value of all a company’s shares of stock. We randomly select 50 stocks from each of the three market capitalization (cap for short) categories: Big market-cap (125 B$ to 922 B$), Mid market-cap (2 B$ to 10 B$) and Small market-cap (300 M$ to 1.2 B$). The stocks from the Big market-cap category are normally the most stable ones relative to the Small-cap stocks, which have the most volatility. Historical data for four market indexes, S&P500 (GSPC), Dow Jones Industrial Average (DJI), NASDAQ Composite (IXIC), and Russell 2000 (RUT), were also included in this study. The data was transformed into matrices with different sizes as explained in next section. In each case, the daily price value for next 10 days are predicted and the estimation methods are compare based on their out-of-sample performance.

### Constructing data matrix

The daily stock price data is transformed into a matrix with *K* rows, samples of vector data, each of length *N*. We get that by stacking *K* rows (*K* samples), each one time shifted from the previous one, all in one big matrix, called the Hankel matrix.

More precisely, the Hankel matrix for this problem is constructed in the following format:

$$\begin{array}{c} {}[\begin{array}{c} t_{1}\\ t_{2}\\ \vdots \\ t_{K} \end{array}]=[\begin{array}{cccc} P(1) & P(2) & \cdots & P(N)\\ P(2) & P(3) & \cdots & P(N+1)\\ \cdots & \cdots & \cdots & \cdots \\ P(K) & P(K+1) & \cdots & P(K+N-1) \end{array}], \end{array}$$

where *P*(*i*) represents the price for day *i*. This is our matrix of data, before normalization and centering.

We first normalize each row (observation) by *Q*th entry, as described earlier, and then subtract the average vector $\bar{x}$ from each row. The prediction is done using the processed data. After doing the prediction, we add back the average vector ${\bar{x}}_{N-M}$ (last *N* − *M* components of $\bar{x}$) from days *M* + 1 through *N* and also multiply the result by the value of *Q*th that was used for normalizing to get back to actual stock prices. We tested different values for *Q* in terms of MSE and estimation variance. For the purpose of this study, we chose *Q* = *M* because it shows the best results in this setting. Recall that *x*_*i*_(*M*) = 1. This column is removed from the data matrix because it does not provide any information. From now on matrix *X* represents normalized and centered price data.

To account for the nonstationarity of the covariance, we use an exponential averaging method as mentioned before. For this purpose, *γ* = 0.98 was selected and the weights smaller than 10^−3^ were considered zero. Then the sample covariance matrix is calculated as

$$\begin{array}{c} \Sigma_{xx}=(\frac{1-\gamma }{1-\gamma^{k+1}})X^{{}^{\prime }}\text{diag}\left(\gamma^{0},\gamma^{1},\cdots ,\gamma^{k}\right)X, \end{array}$$ (21)

where diag(*γ*^0^, *γ*^1^, ⋯, *γ*^*k*^) is a diagonal matrix with (*γ*^0^, *γ*^1^, ⋯, *γ*^*k*^) as the diagonal elements.

We obtained end-of-day stock prices for General Electric and converted this time series into Hankel matrices with different lengths as described above. 2000 samples were used to evaluate the out-of-sample performance of the methods. The values corresponding with the performance metrics presented in this section converge after a few hundred samples. We construct data matrices with 9 different sizes, *M* from 50 to 530 with a 60 day interval, to investigate the effect of length of observation vector on performance.

**Fig 2:**
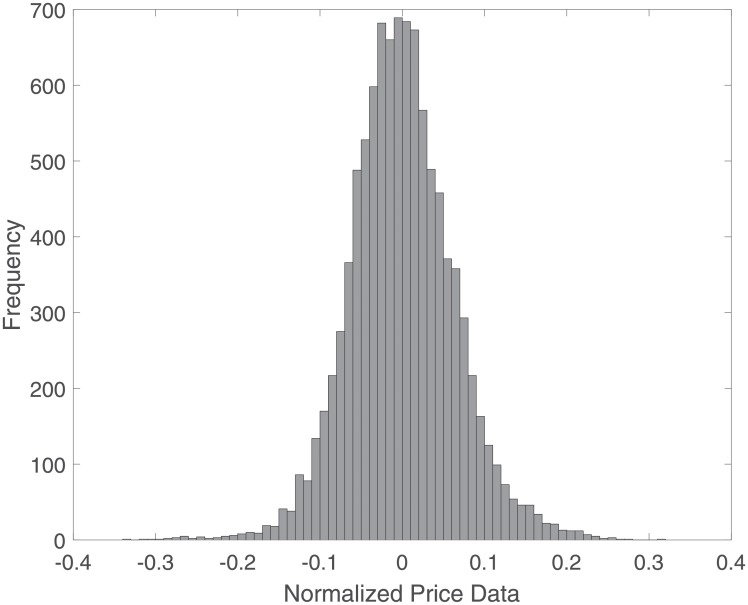
Histogram graph for normalized data.

Fig 2 shows the histogram of normalized data as a representation of the distribution of normalized data; the curve resembles a bell shape.

### MSE performance

Three different estimation methods are implemented for each of the data matrices constructed above. The goal is to predict future price values for the next 10 days (days *M* + 1 to *N*). when it comes to reduced-dimension method, for each *M* we try different values of *L*, the number of principle components. The general goal, as mentioned above, is an estimation technique that has a similar behavior as an ideal Gauss-Bayes estimator but does not have the associated calculation difficulties resulting from ill-conditioning.

We use General Electric price data to calculate the values illustrated in this section. We calculate the squared error (SE) for 2000 samples to evaluate the performance of the methods. We implement our reduced-dimension technique for different *M*s, and for different numbers of principal eigenvalues, *L*.

**Fig 3:**
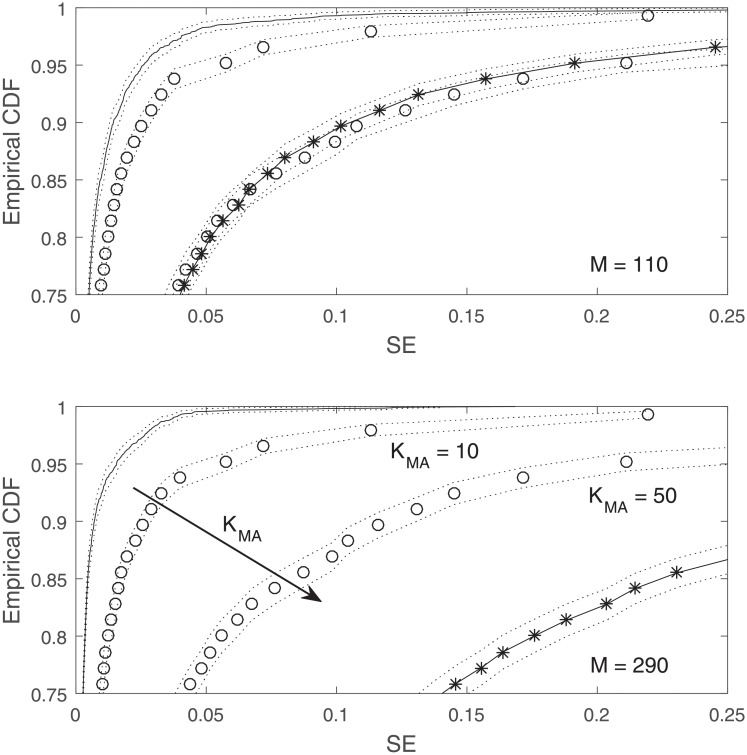
Empirical CDF of SE corresponding to M = 110 and 290. MA_20_ and MA_50_: −*o*−, GB: −*−, RD: Solid lines. Dashed lines illustrate a two standard deviation confidence interval. Plots toward the top and left represent better performance.

Fig 3 shows the empirical Cumulative distribution function (CDF) of the SE for 2 different values of *M*, together with two-standard-deviation confidence interval. Note that to make our comparisons fair and meaningful, we normalized the results from the moving average predictors so that their values are equally normalized with the values from our RD method. When it comes to out-of-sample performance, the numerical complications compromise the estimation accuracy of Gauss-Bayes, causing the SE values for this method to become even worse than the SE plot for the moving average estimators. As we can see, in both plots, our reduced-dimension method is superior to the other two methods. For *M* = 110 some lines are relatively close together. As *M* gets larger, the plot for the reduced-dimension method improves and the plot for Gauss-Bayes gets worse.

Another point worth mentioning is that although adding more data improves the performance of our proposed method, that is not the case for the moving average estimator. As the arrow on the plot on the bottom indicates, by adding more data, moving from $z_{MA_{10}}$ to $z_{MA_{50}}$, the performance of the moving average estimator deteriorates. This behavior is expected since the moving average relies on the momentum, in contrast to the reduced-dimension method, which extracts the essence of the information by projecting onto a smaller subspace.

**Fig 4:**
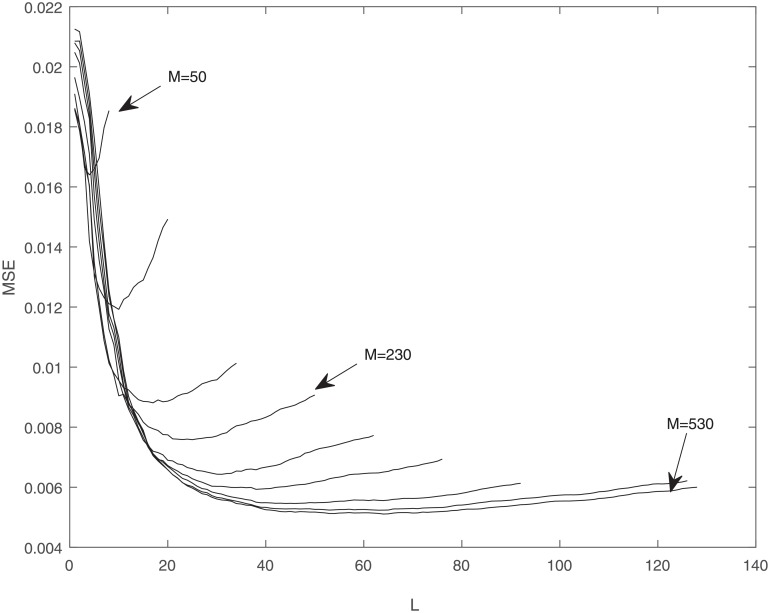
MSE versus *L* in the normalized domain for different *M*s.

Fig 4 shows the values of MSE over all days of estimation versus the value of *L*, for 9 different *M*, lengths of observation vector, from 50 to 530. As we can see, the MSE value is insensitive to the value of *L* for sufficiently large *L*. For small values of *L*, the MSE values fall quickly, but then eventually increase. So if we have a particular constraint on the condition number, we do not lose much in terms of MSE by choosing a reduced-dimension subspace, which leads to a better conditioned problem. After a certain point, adding more data is actually adding noise and the MSE values get worse.

The metric we are looking for is the sum of MSE values over all days of estimation.

For each length of *M*, the values for MSE are captured based on different constraints of the condition number of Σ_*ww*_. The MSE values in the reduced-dimension method are significantly smaller relative to the other two methods.

**Fig 5:**
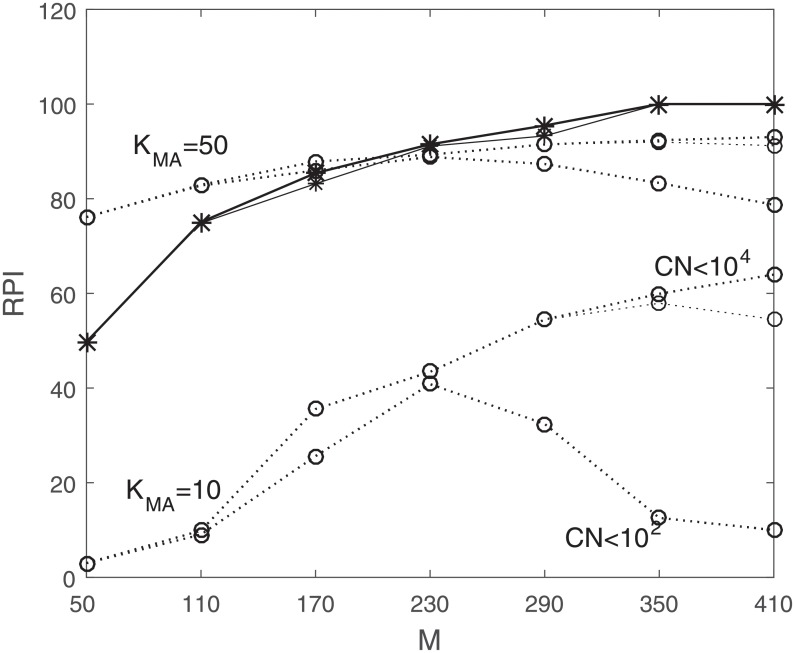
RPI values, subject to different upper limit on condition number of Σ_*ww*_, in each case 10^2^ associated with the line on the bottom, to 10^4^ associated with the line on top, *RPI*_*MA*_: ‥*o*‥, *RPI*_GB_: −*−. Higher plots represent worse relative performance (relative to RD).

Fig 5 shows the relative percentage of improvement (RPI) in the reduced-dimension method compared to the other two methods, calculated as

$$\begin{array}{c} RPI_{\text{GB/MA}}=\frac{-100(MSE_{\text{RD}}-MSE_{\text{GB/MA}})}{MSE_{\text{GB/MA}}}. \end{array}$$ (22)

Note that since the denominator in the equation is *MSE*_GB/MA_, the improvement percentage does not exceed 100% but the actual MSE values are further apart in absolute terms than illustrated here. For example for *M* = 350, the MSE value for reduced-dimension is between 0.0052 to 0.018, while the MSE in Gauss-Bayes is around 6.33 × 10^6^. The three (overlapping and therefore appears as only a single plot) lines on top (-*-) of Fig 5 compare the reduced-dimension to Gauss-Bayes (*RPI*_*GB*_). The three lines on top (‥o‥) correspond to the comparison of the reduced-dimension and moving average ($RPI_{MA_{50}}$) and the three lines on the bottom (‥o‥) correspond to ($RPI_{MA_{10}}$). In each case the three lines are subject to different upper limits on the condition number (10^2^, 10^3^, and 10^4^). It is worth mentioning that the condition number of Σ_*yy*_ starts from 10^3^ for *M* = 50 and goes up to 10^19^ for *M* = 530. The upper limit on the condition number of Σ_*ww*_ changes from 10^2^, associated with the lines on the bottom in each case, to 10^4^, the lines on top, for all values of *M*.

In general, by increasing *M*, more information is available in each observation, resulting in better performance of the prediction in terms of smallest MSE values. This can be observed easily in the RPI plots in Fig 5 in comparison to the moving average cases since the MSE values in the those cases are almost constant for different values of *M*. The percent of improvement of MSE values corresponding to the reduced-dimension method increases as *M* increases. This is as expected since more information is available in each observation, resulting in better performance. However after a certain point the RPI flattens out suggesting adding more data at this point is increasing the noise and does not improve the performance.

As we can see, in some cases there is a slight decrease in the improvement rate of the reduced-dimension method compared to the moving average method. A possible explanation for this observation is that when we fix some constraint on condition number, we are actually limiting the value of *L*, and by increasing *M*, after a certain point, we mostly increase the noise, and the MSE value gets worse, which is consistent with Fig 4. Table 1 shows the average RPI values for all stocks in different market-cap categories and average RPI values for market indexes. The reduced-dimension method consistently shows better performance than the other two methods.

**Table 1:** Average RPI values for stocks in different market-cap categories and average RPI values for market indexes (*M* = 350).

MSE	*RPI*_*GB*_	$RPI_{MA_{10}}$	$RPI_{MA_{50}}$
Small-Cap	100%	51%	88%
Mid-Cap	100%	54%	88%
Big-Cap	100%	56%	89%
Market indexes	100%	55%	88%

Matlab’s two-sample t-test function was used to determine the MSE values from our proposed method for 50 stocks in each market-cap category is significantly smaller than the average of the MSE values generated for the same sample using other methods at 5% significance level (*α* = 0.05). When *p* < *α* and *h* = 1, the null hypothesis that the two samples have the same mean is rejected, concluding that the difference between the averages of the two sets of samples is statistically significant at *α* significance level. As shown in Table 2, the results indicate that the average of the MSE values for predictions from our method is significantly smaller than the average of MSE values from other competing methods at 0.05 significance level.

**Table 2:** Statistical analysis for MSE values for stocks in different market cap categories (*M* = 350).

T-test	against	*MSE*_*GB*_	$MSE_{MA_{10}}$	$MSE_{MA_{50}}$
Small-Cap	p-value	0.0024	0.0075	0.00068
	h	1	1	1
Mid-Cap	p-value	0.0283	0.0066	0.000038
	h	1	1	1
Big-Cap	p-value	0.0021	0.00048	0.00001
	h	1	1	1

**Fig 6:**
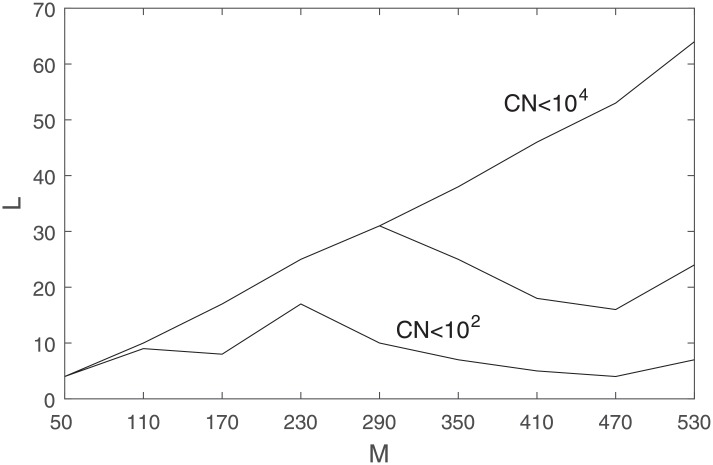
Best *L* corresponding to best MSE values subject to different limits on condition number, 10^2^ associated with the line on the bottom, 10^3^ associated with the line in the middle, and 10^4^ associated with the line on top.

Recall that *L* represents the number of eigenvalues required from the diagonal matrix *S* to represent the bulk part of the information carried in *x*. Fig 6 investigates the dimension of the target subspace by plotting the value of *L* corresponding to best MSE for different *M*s, subject to different limits on condition number (the same case as in Fig 5).

As the upper limit on condition number increases, the value of MSE improves as *M* increases, and we need a bigger subspace, bigger *L*, to extract the information. However, as the bottom three plots in Fig 6 show, the value for best *L* flattens out after a certain point.

### Directional change statistic performance

**Fig 7:**
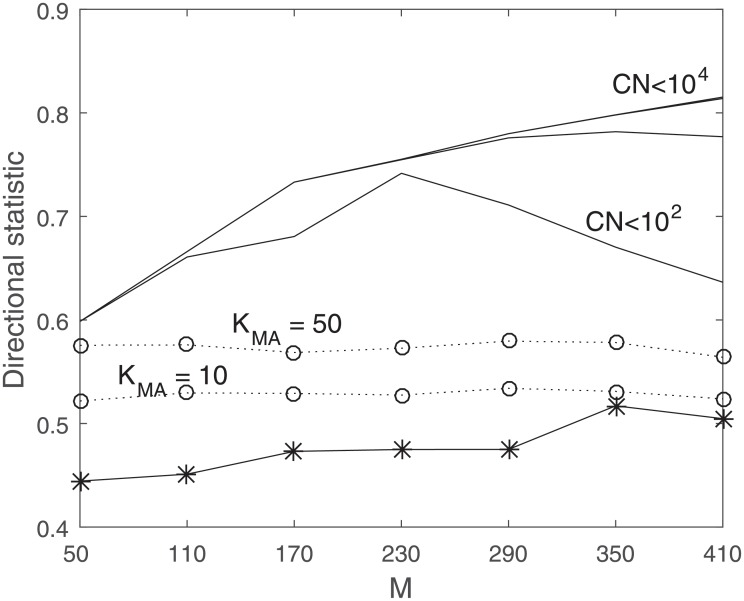
Best directional statistics subject to different upper limit on condition number of Σ_*yy*_, 10^2^ associated with the line on the bottom, to 10^4^ associated with the line on top, GB: −*−, RD: Solid lines, MA: ‥*o*‥ (MA_10_ on the bottom and MA_50_ on top). Higher plots represent better performance.

The other evaluation metric that we are interested in is the directional statistic which measures the matching of the actual and predicted values in terms of directional change. Fig 7 shows the average directional statistic over 10 days of estimation using the same *K* = 2000 samples. As the plot indicates, the reduced-dimension method is superior in terms of directional change statistic. It is interesting to note that the directional statistic improves as *M* increases, and then eventually flattens out, consistent with previous plots.

Table 3 shows the average value for directional statistic for stocks in different market cap categories and indexes for *M* = 350 for Σ_*ww*_ condition number limited to 10^4^. The reduced-dimension method is superior to the other two methods in terms of directional change estimation. It is important to note that the values represented in Table 3 are associated with a specific *M* for all companies. In practice, it is recommended to tailor the value of *M* for each company to get the best results.

**Table 3:** Average directional statistics for stocks in different market cap categories (*M* = 350).

Directional Statistic	MA_10_	MA_50_	GB	RD
Small-Cap	0.56	0.61	0.51	0.78
Mid-Cap	0.58	0.62	0.51	0.79
Big-Cap	0.60	0.66	0.51	0.80
Market Indexes	0.63	0.70	0.50	0.79

Matlab’s two-sample t-test function was used to determine if the average of the directional statistics from our method for 50 stocks is significantly larger than the average of directional statistics from other methods. Table 4 lists the p-value and h-statistic for each test. The results also indicate that the average of directional statistics from our method is significantly larger than the average of the directional statistics from other competing methods at 5% significance level.

**Table 4:** Statistical analysis for directional statistics values for stocks in different market-cap categories (*M* = 350).

T-test	against	*D*_*GB*_	$D_{MA_{10}}$	$D_{MA_{50}}$
Small-Cap	p-value	<10^−10^	<10^−10^	<10^−10^
	h	1	1	1
Mid-Cap	p-value	<10^−10^	<10^−10^	<10^−10^
	h	1	1	1
Big-Cap	p-value	<10^−10^	<10^−10^	<10^−10^
	h	1	1	1

### Volatility

Another important parameter that we estimate is the volatility of the prediction, measured in terms of its standard deviation. The square root of the diagonal elements of the estimated covariance, ${\hat{\Sigma }}_{zz}$, are the estimated standard deviations for individual days of estimation. The estimate of the covariance in each method is

$$\begin{array}{cc} {\hat{\Sigma }}_{GB} & ={\hat{\Sigma }}_{z|y}=\Sigma_{zz}-\Sigma_{zy}\Sigma_{yy}^{-1}\Sigma_{yz},\\ {\hat{\Sigma }}_{RD} & ={\hat{\Sigma }}_{z|w}=\Sigma_{zz}-\Sigma_{zw}\Sigma_{ww}^{-1}\Sigma_{wz}, \end{array}$$ (23)

**Fig 8:**
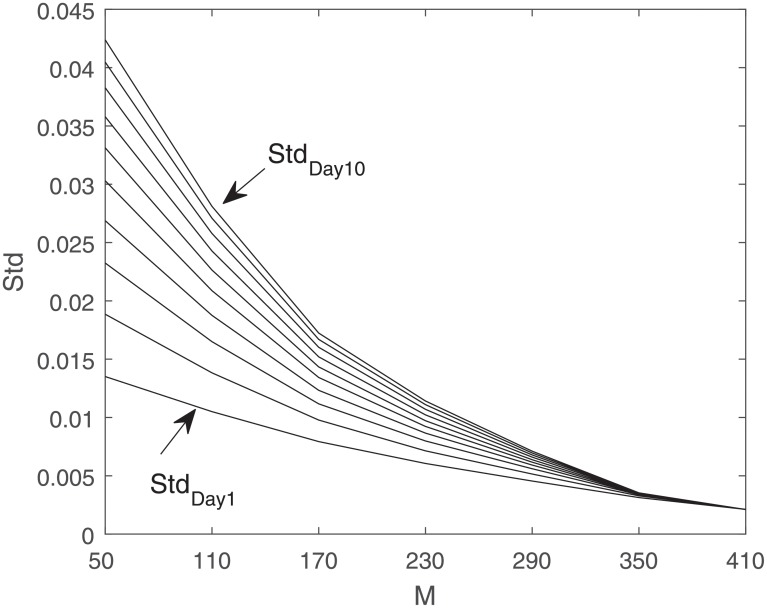
Standard deviation of individual days of estimation, RD: Solid line.

However, note that because of the poor conditioning of Σ_*yy*_, using the formula above for Σ_*GB*_ has numerical issues. Hence, we omit their values here. In general the standard deviation values increase moving from day 1 to day 10 of prediction, since less uncertainty is involved in the estimation of stock prices of days closer to the current day. In Fig 8, the standard deviation for individual days of estimation, days 1 to 10, are plotted versus *M*, the length of observation vector, for the reduced-dimension method. In the reduced-dimension method, the standard deviation values decrease as *M* increases because more information is provided in each observation. For sufficiently large *M*s, the standard deviation values for different days are very close.

## Conclusion

In this paper we introduced a new method for predicting future stock price values based on covariance information. We develop this method based on a filtering operation using principle components to overcome the numerical complications of conditional mean. We also introduced a procedure for normalizing the data. The matrix of data was constructed in different sizes to investigate the effect of length of observation vector on prediction performance. Our method has showed consistently better out-of-sample performance than Gauss-Bayes (multivariate conditional mean), a numerically challenged estimator, and moving average, an easy to use estimator, for 5 different companies in terms of mean squared error and directional change statistic.

The proposed method can be modified to include multiple predictors. The significance of the proposed approach will be even more apparent when using multiple predictors because where observation vectors are longer it becomes almost impossible to rely on conditional mean due to the severe ill-conditioning of the covariance matrix.

## Supporting information

S1 File (XLSX)
